# Reliving the Old Dream: Rural Tourism Autobiographical Memory on Behavioral Intention

**DOI:** 10.3389/fpsyg.2022.736637

**Published:** 2022-04-06

**Authors:** Zhifeng Zhao, Zhiwei Li, Cai Chen

**Affiliations:** ^1^School of Tourism, Hainan Normal University, Haikou, China; ^2^School of Tourism and Service, Chongqing University of Education, Chongqing, China

**Keywords:** autobiographical memory, memory rehearsal, memory impact, place attachment, revisit intention, recommendation intention

## Abstract

This paper evaluates a theoretical model based on hypothesized relationships among four constructs, namely, autobiographical memory (memory rehearsal and memory impact), and place attachment as antecedents of revisit intention and recommendation intention in the context of rural tourism in China. The results of 301 Chinese tourists show that the two dimensions of tourists’ autobiographical memory (memory rehearsal and memory impact) affect the tourists’ intention to revisit and recommend. Place attachment plays an intermediary role among tourists’ autobiographical memory, revisit intention, and recommendation intention. This study is the first to apply the structural dimension of autobiographical memory has been applied to rural tourism in China. Theoretical and managerial implications are discussed based on the study results.

## Introduction

By the end of 2019, the total number of Chinese population at the mainland reached 1,400.05 million, urban permanent residents numbered 848.43 million, accounting for 60.60% of the total population (the urbanization rate of permanent residents) (National Bureau of Statistics of China, 2020). Under the background of rapidly promoting urbanization, a large number of urban residents want to escape from urban life, the countryside has become synonymous with “poetically habitat.” According to statistics, the national leisure agriculture and rural tourism received about 3 billion tourists in 2018, with an operating income of more than 800 billion Yuan (National Bureau of Statistics of China, 2019). It can be seen that rural tourism can stimulate the economic growth of tourism destinations, which is an important driving force of rural development in China. However, there are high substitutability (Tang et al., 2011) and serious homogenization (Lu and Cao, 2008) among rural tourism destinations, which leads to intensified competition among rural tourism destinations. Therefore, it is particularly important to cultivate tourists’ loyalty to rural tourism destinations (Chi and Qu, 2008; Campón-Cerro et al., 2017). As the main attribute of tourist behavior or loyalty (Yoon and Uysal, 2005; Nguyen et al., 2019) and the reflection of tourist satisfaction (Kaur and Kaur, 2019), revisit intention and recommendation intention are also becoming more and more important. At the same time, relationship marketing also advocates competitive advantage through revisit and recommendation (Shirazi and Som, 2011). In view of this, post-travel behavior intention of tourists is considered as a major focus to improve the competitiveness of rural tourism destinations (Campón-Cerro et al., 2017; Ryglová et al., 2018) and promote the sustainable success of rural tourism.

In the marketing literature, the purchasing decision-making process of consumers involves multiple stages, among which the post-purchase behavior is an important stage (Westbrook, 1987). Many studies have shown that the post-purchase behavior of customers is an explicit behavior triggered by satisfaction (Kotler and Armstrong, 2001), and the cost of retaining existing customers is much lower than the cost of acquiring new ones (Nigel and Jim, 2002). Similarly, the behavior of tourists also involves three stages: before, during, and after tourism (Zhao and Yang, 2017). Among them, the post-travel behavior is also called the future behavior, which not only represents the end of the purchase process, but also directly affects the next purchase decision (Westbrook, 1987). When tourists’ expectations are met and they are satisfied with the destination, there is often a revisit or recommendation intention to the destination (Kaur and Kaur, 2019). For destinations, revisiting means higher incomes and returns (Darnell and Johnson, 2001) and opportunities to build long-term relationships with tourists (Petrick, 2004). In addition, returning visitors are also “word-of-mouth agents,” who advertise destinations to other potential tourists (Shoemaker and Lewis, 1999) and establish a positive image of the destination (Pritchard, 2003) through recommendations from family, friends, and past tourists who have visited are an important source of information for potential tourists to choose their destination (Jalilvand et al., 2017), thereby reducing the marketing costs of travel companies (Haywood, 1989). Previous studies have shown that many rural tourism destinations rely heavily on the post-travel behavior of tourists, with tourists being able to revisit or recommend destinations to other potential tourists, such as friends or relatives (Kozak, 2001; Bowen and Shoemaker, 2003), which can provide a steady stream of income to travel destinations (Darnell and Johnson, 2001; Hong et al., 2009). Therefore, the intentions to revisit and recommend tourists represent the basic goals of tourism destinations (Vidič et al., 2019), which is also the problem considered by tourism scholars.

Although researchers have recognized the importance of the decision-making and behavior of tourist revisiting and recommendation and have examined relevant antecedent variables, including destination image (Chi and Qu, 2008; Stylos et al., 2016), satisfaction (Petrick et al., 2001; Lee et al., 2019), perceived value (Scaglione and Mendola, 2016), service quality (Frochot and Hughes, 2000; Mai et al., 2019), travel experience (Kim et al., 2012; Kim, 2017), and so on. According to the forward frame theory, the previous information, as a memory model, affects the cognitive of the current experience (Bower et al., 1979; Alba and Hasher, 1983). Studies have shown that an individual’s experience at a destination directly affects his or her future decision-making on destination selection and visit (Pearce, 1982; Chon, 1991; Chon and Olsen, 1991; Ross, 1993). There is a link between prior experience and destination loyalty (Lehto et al., 2004), remembering that positive experiences will enhance the intention to revisit and recommend (Oh et al., 2007; Williams and Soutar, 2009; Hosany and Witham, 2010; Miao et al., 2014; Coudounaris and Sthapit, 2017). In other words, some tourism studies have recognized the role of personal memories in influencing behavioral intentions (Bartoletti, 2010; Martin, 2010; Marschall, 2012a). Therefore, it is very important for rural tourism destinations operators and managers to understand how tourists create and recall their memories (Tung et al., 2016). However, so far, few people have paid attention to the relationship between tourist autobiographical memory and post-trip behavioral intention (such as revisit and recommendation), especially from the dimension of autobiographical memory to explore these factors on the formation of post-purchase decisions of tourists are rarely studied. In addition, the relationship between people and places has been conceptualized as place attachment (Moore and Graefe, 1994; Williams and Vaske, 2003; Scannell and Gifford, 2010), and the links between place attachment and destination loyalty and revisit intention have been revealed in the tourism literature (Lee et al., 2012; Prayag and Ryan, 2012; Isa et al., 2019). However, how tourist autobiographical memories can trigger positive intentions to revisit and recommend through place attachments is rarely discovered. Therefore, in order to better understand what drives tourist post-travel behavioral intentions and provide important management inspiration for rural tourism destination managers and marketers, this study aims to explore the relationship between tourist autobiographical memory, place attachment, and tourist post-travel behavioral intentions. This provides destination managers and marketers with a comprehensive understanding of how tourists create and recall their memories to help them establish management and marketing strategies.

## Literature Review and Research Hypothesis

### Tourist Autobiographical Memory and Place Attachment

Memory is the reflection of the human brain on the things you have perceived, the problems you have thought about, the emotions you have experienced, and the actions you have done. Long-term memory, an important basis for the accumulation of individual experiences and the development of cognitive abilities, is thought to be the most interesting (Larsen, 2007). Semantic memory and episodic memory are two basic forms of information stored in long-term memory (Shao, 2013). Autobiographical memory is a more special kind of episodic memory, which is a personal memory of his or her past life experience and behavior (Araújo et al., 2019), which comes from personal life experience. Autobiographical memory is not only related to specific events, but also an important part of self-identity, which affects the formation of personal history and self-concept (Fivush, 2011). The memory of a visitor’s personal experience is considered to be an autobiographical memory (Kim and Jang, 2014).

The relationship between tourism and memory is a complex and multidimensional problem, which deserves more and more scholars’ attention. Compared with the most aggressive advertising, memory may be a determining factor in people’s travel choice before they travel, and once they arrive at their destination, memory may have a strong impact on the travel experience. After the trip, memory affects tourist perception of short-term and long-term experiences again (Marschall, 2012a). In fact, tourism helps people construct stories and collect memories (Kahneman, 2011), and memorability is considered to be an important result of the travel experience (Pine and Gilmore, 1998; Chhetri et al., 2004). For this reason, Marschall (2012a) proposed “Personal Memory Tourism,” which was considered as a form of tourism driven by one’s past memories. As a result, for the study of tourism experience, some scholars have shifted their attention to the connection between memory and experience. In particular, they emphasized memorable tourism experiences (Tung and Ritchie, 2011; Kim et al., 2012; Chandralal and Valenzuela, 2013; Kim, 2014; Kim and Ritchie, 2014; Zhang et al., 2018). According to Kim and Chen (2019), memorable travel experiences are highly self-centered and are considered to be a particular subjective event in life, which is stored in long-term memory as part of autobiographical memory. However, as far as we know, flashbulb memories, which are extremely vivid and lasting memories of major events, are the main ones mentioned in memorable travel experiences (Larsen, 2007). From the perspective of cognitive psychology, autobiographical memory includes four latent constructs, recollection, belief, impact, and rehearsal (Fitzgerald and Broadbridge, 2013). Among them, memory impact refers to the properties of significance, emotional intensity, and consequences of memory events on individuals. Meanwhile, memory rehearsal is defined as the “frequency with which an event is recalled, either personally or interpersonally, whether voluntarily or involuntarily” (Fitzgerald and Broadbridge, 2013). Some studies have verified the robustness of the two basic structures of memory rehearsal and memory impact in tourism research (Jorgenson et al., 2019). Therefore, we assessed personal autobiographical memory based on the intensity of their recall of events and the frequency with which they were recalled.

Tourism is an important way and method for people to perceive and understand the environment. A tourism destination is not only a place of consumption, but also has profound and sustainable significance for tourists (Fuhrer et al., 1993; Williams and McIntyre, 2012). Since memory is a positive construction process, where information is captured, stored, and then extracted for decision making (Braun, 1999). Autobiographical memory connects all kinds of things encountered by tourists with their destination, which has a continuous impact on tourists. The experience you remember (long-term memory) may be a better predictor of repetitive experiences in the future (Agapito et al., 2017). In particular, autobiographical memory plays an important social functions. By sharing the past travel experience with others, tourists can have an emotional resonance with others and establish a close relationship (Alea and Bluck, 2003).

Research has shown that memory is an important component of place attachment, and place attachment relies on positive memorable experiences (Hammitt et al., 2006). When the tourist’s positive memory is awakened, the tourist destination may be transformed into a place with emotional and symbolic significance (Yin et al., 2017). The aspect of the psychological process dimension of place attachment is the cognitive and affective level, autobiographical memory deepens the relationship between tourists and their destinations. Based on the above reasoning, this paper suggests that memory impact and memory rehearsal may help strengthen place attachment and promote an emotional connection between people and places. As a result, it is assumed that:

**Hla:** Memory rehearsal has a positive effect on place attachment.
**Hlb:** Memory impact has a positive effect on place attachment.

### Place Attachment and Post-travel Behavior Intention

With the emotional turn of geography, place is no longer a cold space, but is endowed with meaningful space by human beings. Human space experience is an important way to construct and explain place. Place is not only a geographical phenomenon, but also contains human experience (Gregory et al., 2017). As the basic space unit carrying “man-land” activities, place carries the rich emotional experience of human beings. Place attachment involves “the interaction of emotions, knowledge, beliefs, and behaviors” (Low and Altman, 1992, p. 5), in which emotion is the most important component (Low and Altman, 1992). Scannell and Gifford (2010) synthesize place attachment into a three-dimensional model of “person-process-place.” The person dimension of place attachment refers to its individually or collectively determined meanings. The psychological dimension includes the affective, cognitive, and behavioral components of attachment, while the place dimension emphasizes the description of the spatial scope, physical, and social characteristics of the place.

In the literature of environmental psychology, place attachment refers to the emotional and psychological bonds that exist between an individual and a specific environment (Williams et al., 1992). In addition to place attachment to the usual environment, tourists can also develop an attachment to the unusual environment, which reflects the emotional connection of tourists to the tourist destination. Place attachment includes cognitive, affective, and behavioral elements, the core of which is the emotional experience between people and places (Hummon, 1992; Scannell and Gifford, 2010). Cognitive processes elaborate the importance of place-related memories, knowledge, beliefs, and meaning, which have formed an inherent schema incorporated into the self-concept. On the basis of these cognitions, places can awaken positive emotions and bring people belonging and happiness. There will be a deeper emotional connection between people and places. Under the influence of cognition and affection, the behavior of place attachment is approaching. The results show that place attachment is an effective variable to predict tourist satisfaction and loyalty to their destination during holiday travel (Yuksel et al., 2010). Individuals with higher place attachment are also more likely to revisit the place in the future, and the more loyal they are (George and George, 2004).

Post-travel behavioral intention refers to the more rational psychological tendency of tourists after traveling. In this study, behavioral intention indicates the possibility of returning and recommending the place in the future holidays. As a driving factor of specific future behaviors, place attachment is an important antecedent variable that affects tourist behavior (Lee and Shen, 2013). Thus, place attachment has recently been used as a key factor in understanding the decision-making process of tourists (Hwang et al., 2005). Since place becomes the center of personal meaning, under the influence of self-enhancement motivation, tourists are more likely to revisit and recommend the destination to others. Based on the above reasoning, the following hypothesis is proposed:

**H2a:** Place attachment has a positive effect on revisit intention.
**H2b:** Place attachment has a positive effect on recommendation intention.

### The Intermediary Role of Place Attachment

According to the above reasoning, there is a corresponding logical relationship among tourist autobiographical memory, place attachment, and behavior intention. Memory is the ability to store and retrieve information, and it acts as a controller of acquired knowledge, behavior, and consumer preferences (Roediger and Wertsch, 2008). Tourism experience is stored in our memory for later use (Staresina and Davachi, 2009), which affects consumer preferences and decision-making behavior (Chandralal et al., 2014; Tsai, 2016). Therefore, tourist satisfaction and revisit intention are highly correlated with their tourism experience (Otto and Ritchie, 1995; Ramires et al., 2018). Memory is considered to be the most important source of information that affects tourist revisit intention (Braun-LaTour et al., 2006). Studies have shown that tourist previous visits or past experiences enhance their knowledge of the destination and increase their chances of recommending and revisiting the destination in the future (Kozak, 2001; Huang and Hsu, 2009; Chen and Chen, 2010). Semrad and Rivera (2018) found that a memorable tourism experience has a significant positive impact on recommendation intention and revisit intention (Barnes et al., 2016), and tourism experience influenced loyalty through satisfaction and memory (Manthiou et al., 2015). These studies further confirmed the effect of memory on future behavior. It can be seen that memory is a key factor to choose the destination (Solisradilla et al., 2019).

Generally speaking, post-travel behavioral intention is often measured by revisit intention and recommendation intention (Chi et al., 2013). Therefore, post-travel behavioral intention can be regarded as the agent of loyalty. Place attachment is the central to tourist future behavior and intention (Neuvonen et al., 2010; Prayag and Ryan, 2012), some places of special significance may induce a person’s actual behavior in life (Vaske and Kobrin, 2001; Kyle et al., 2003) or even drive loyalty behavior (Prayag and Ryan, 2012; Lee and Shen, 2013). Studies by scholars have found that the satisfying experience of previous visits confirms the tourist attachment to the destination, which further motivates them to return (Petrick et al., 2001; George and George, 2004). Loureiro (2014) emphasizes that memory is related to experience and takes attachment and behavioral intention as results.

In addition, we have also noticed that autobiographical memory has the instructive function, which can guide, motivate, and inspire individual behaviors and instruct individuals how to seek advantages and avoid disadvantages (Pillemer, 2003), which is potentially related to whether tourists will revisit the destination (Jorgenson et al., 2019). The literature shows that positive destination memory is the basic determinant of post-travel behavior (Marschall, 2012b). When the autobiographical memory of tourists is awakened, their subsequent behaviors, attitudes, and beliefs will be affected by it. In particular, it enhances tourist identity or strong attachment to a place (Oh et al., 2007; Martin, 2010; Kim et al., 2012; Chandralal and Valenzuela, 2013). Place attachment involves an interplay of affect and emotions, knowledge and beliefs, and behaviors and actions (Kyle et al., 2014).

Autobiographical memory lays more emphasis on the emotional attributes and influences of memory, which may enhance the emotional connection between tourists and their destinations, and then affect their behavioral intentions. More importantly, these memories can affect place attachment to the destination, intention to revisit and recommend, and even sharing post-travel experiences with their family and friends (Martin, 2010). It can be seen that place attachment affects tourist behavior intention (Lee and Shen, 2013) and plays an intermediary role between tourism attitude and behavior intention (Tsai, 2012). Therefore, this paper argues that place attachment is a key variable of rural tourism. The autobiographical memory is believed to be a positive driver of the affective association with place, and place attachment can in turn induce future travel behavior. Therefore, the hypothesis is put forward:

**H3a:** Place attachment played a mediating role in the relationship between memory rehearsal and revisit intention.
**H3b:** Place attachment played a mediating role in the relationship between memory rehearsal and recommendation intention.
**H3c:** Place attachment played a mediating role in the relationship between memory impact and revisit intention.
**H3d:** Place attachment played a mediating role in the relationship between memory impact and recommendation intention.

### Conceptual Model

**FIGURE 1:**
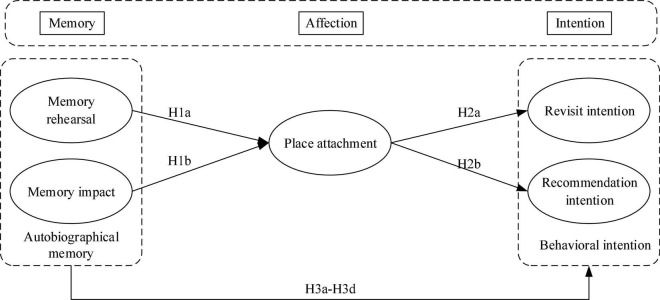
Conceptual model.

Based on the above discussion and hypothesis, we propose a conceptual model (Figure 1). In this model, memory rehearsal and memory impact are the perception of tourist experience, which will affect tourist attachment to their destination, and then affect tourist future decision-making, including revisit intention and recommendation intention. The theoretical basis of this model is the “cognition-affection-behavior” (CAB) model proposed by Lavidge and Steiner (1961).

In our model, parts of tourist autobiographical memory represent cognition, parts of tourist autobiographical memory and place attachment represent affection, revisit intention and recommendation intention represent behavior, and the relationship among these constructs also follows the CAB hierarchy. This model reflects the fundamental psychological process of human beings (Lavidge and Steiner, 1961; Baloglu, 1998). As shown in Figure 1, this framework not only illustrates the relationship between variables, but also illustrates how autobiographical memory influences the future behavioral intentions (revisit and recommendation intentions) through place attachment, namely, “memory-affection-intention.”

## Research Method

### Variable Measurement

In order to test the proposed hypotheses and conceptual model, this study needs to measure the following four constructs: autobiographical memory, place attachment, recommendation intention, and revisit intention. Autobiographical memory is based on a previous measurement scale (Talarico et al., 2004; Jorgenson et al., 2019), including two dimensions and nine items. The measurement of place attachment was adapted from Kyle et al. (2005), consisting of five items. The measurement of recommendation intention was modified from Zeithaml et al. (1996) and Kim et al. (2011), which consists of four items. The scale of revisit intention was based on Hung et al. (2014), including three items. All the items were measured using a five-point Likert scale (1 = strongly disagree to 5 = strongly agree). Respondents were asked to rate items from strongly disagree to strongly agree. The above scales are detailed in Table 1.

**TABLE 1:** Assessment of measurement model.

Items	Mean	Standard loadings	Composite reliability	AVE	Cronbach’s α
**Memory rehearsal**			0.834	0.627	0.753
Since it happened, I have talked about this event.	5.096	0.790			
Since it happened, I have thought about this event.	5.196	0.728			
This memory has previously come to me “out of the blue,” without my trying to think about it.	4.983	0.862			
Since it happened, I have written about this event to others (e.g., wechat, email, blog, letter, and text).*	4.844				
As I remember the event, I can feel now the emotions I felt then.*	5.336				
**Memory impact**			0.755	0.509	0.852
As I remember the event, it comes to me in words or in pictures as a coherent story or episode and not as an isolated fact, observation, or scene.	5.13	0.650			
This memory is significant in my life because it imparts an important message for me or represents an anchor, critical juncture, or turning point.	5.08	0.756			
This memory has consequences for my life because it influenced my behavior, thoughts, or feelings in noticeable ways.	4.821	0.721			
While remembering the event, the emotions are extremely positive.*	5.17				
**Place attachment**			0.76	0.52	0.739
This rural tourism destination makes a lot to me	5.299	0.682			
I really like this rural tourism destination.	5.236	0.861			
I identify strongly with this rural tourism destination.	5.156	0.594			
I enjoy this rural tourism destination more than any other village.	4.223				
**Recommendation intention**			0.78	0.546	0.771
I will recommend this village to others	5.086	0.593			
I will recommend this village to my friends and family	5.096	0.826			
I will say positive things about this village to others	5.063	0.777			
**Revisit intention**			0.844	0.644	0.802
I think I will come back to this village in near future	4.997	0.787			
I tend to visit this village again	4.801	0.790			
I’d love to come to this village again	4.625	0.830			

*Represents the items deleted in measurement model test.

### Research Areas and Data Collection

Chongqing is located in the southwest of China and in the upper reaches of the Yangtze River, with 31.24 million permanent residents and an urbanization rate of 66.8%. In recent years, with the rapid development of rural tourism and great potential of the tourist market in Chongqing, which occupies an important position in the industry of Chongqing. In 2018, Chongqing has built 12 demonstration counties and 23 demonstration sites for national-level leisure agriculture and rural tourism. There are a total of 14 famous towns for landscape tourism with national characteristics and seven famous villages. Rural leisure tourism in the city received more than 205 million tourists, with a comprehensive tourism income of 67.7 billion yuan (Ministry of Agriculture and Rural Affairs of the People’s Republic of China, 2019). Therefore, Chongqing is chosen as the research area.

The objective of this study is the domestic tourists who have experienced rural tourism in Chongqing. The questionnaire survey was mainly carried out in residential communities, tourist attractions, city squares, and parks in Chongqing. This study collected data from July to August 2019, a team of three students was recruited and trained for this purpose. First of all, ask the respondents if they have any experienced rural tourism in Chongqing. If so, please answer the following questions to identify qualified samples. A total of 360 questionnaires were distributed, 338 points were recovered, invalid questionnaires were removed, 301 questionnaires were recovered, and the effective rate was 86%. In terms of gender, 34.2% were men and 65.8% were women. Most of the respondents (66.1%) were between 18 and 31 years old, the second age group (21%) was 31–50 years old. Most of the respondents (58.8%) had a 4-year university degree, 15.3% of respondents had a 2-year college degree.

## Research Results

### Measurement Model

The reliability analysis was measured by Cronbach’s α coefficient, and the reliability coefficients of all latent variables almost reached above 0.75, indicating a high reliability of the scale. Second, to test the validity, exploratory factor analysis (EFA) was used to test 23 items. With the eigenvalue greater than 1 as the measurement standard, the maximum variance method was adopted, and the factor load greater than 0.4 and the common degree greater than 0.5 as the criterion (Hair et al., 1998), item loading on more than one factor had to differ by ≥ 0.10 in the loading to be retained (Raymond et al., 2010),and five factors were extracted. The cumulative variance contribution rate was 67.340%. According to the results of confirmatory factor analysis (CFA), χ^2^ = 113.569, *df* = 76, χ^2^/*df* = 1.494, RMSEA = 0.041, CFI = 0.979, TLI = 0.971, and IFI = 0.947, which indicated that the overall model was well-adapted (Wen et al., 2004). Convergent validity was measured by factor loadings, composite reliability, and average variance extraction (AVE). All the factor loading was above 0.5, which was consistent with the requirements standards (Hair et al., 1998). The combined reliability of all constructs ranged from 0.755 to 0.844, which meets the recommended standards (Bagozzi et al., 1991). The value of AVE was 0.509–0.644, greater than the required value of 0.5, which met the requirements, indicating that the convergence validity of the model was good (Bagozzi et al., 1991; Table 1). The square root of the AVE of each latent variable was 0.713–0.802, and the correlation coefficient of the latent variable was 0.174–0.451. The square root of the AVE of each latent variable is greater than its correlation coefficient, which shows that the discriminant validity of each variable is better (Fornell and Larcker, 1981; Table 2). In addition, Harman’s single factor method is also used to detect common-method variance of questionnaire data. After the test, it was found that the explanatory variance of the first rotated factor was 33.432%, so there was no problem of significant homologous variance. Meanwhile, a non-response bias test was applied by comparing early (i.e., the first half respondents) and late respondents (i.e., the second half respondents) based on the questionnaire return date. There was no significant difference at the 0.01 significance level, indicating that non-response bias was not the main concern (Armstrong and Overton, 1977).

**TABLE 2:** Means, SD, and correlations among variables.

	Mean	s.d.	1	2	3	4	5
Memory rehearsal	5.092	1.159	0.791				
Memory impact	5.01	1.151	0.244**	0.713			
Place attachment	5.23	1.897	0.246**	0.408**	0.721		
Recommendation intention	5.081	1.095	0.174**	0.364**	0.362**	0.738	
Revisit intention	4.807	1.312	0.280**	0.444**	0.451**	0.431**	0.802

**Means significant at the significance level of 0.01; the diagonal of the matrix is the square root of the average variance extract, and the lower half of the matrix is the correlation coefficient.

### Hypothesis Testing

The maximum likelihood (ML) estimation was used to verify the hypothesis by using the software of Structural Equation Modeling (SEM). The results showed (χ^2^ = 153.8000, *df* = 79, χ^2^/*df* = 1.947, RMSEA = 0.056, CFI = 0.959, TLI = 0.945, IFI = 0.959) that the overall fit of the model was good. The effect of memory rehearsal on place attachment was significant (β = 0.189, *P* < 0.05). H1a hypothesis was established. The effect of memory impact on revisit intention was significant (β = 0.620, *P* < 0.001), and H1b was assumed to be true. The effect of place attachment on revisit intention was significant (β = 0.670, *P* < 0.001), and H2a hypothesis was established. The effect of place attachment on the recommendation intention was significant (β = 0.560, *P* < 0.05). Further analysis showed that 47.8% of the variation of place attachment could be explained by the combined effects of memory rehearsal and memory impact, while place attachment could, respectively, explain 44.9 and 31.4% of the variation of revisit intention and recommendation intention.

The Bootstrap method of SPSS software was used to test the intermediary effect (Hayes, 2013), and the intermediary role of place attachment between autobiographical memory and behavior was tested. The sample size was selected to be 5,000, a 95% CI was set, and the non-parametric percentile method with deviation correction was used for Bootstrap sampling. The results showed that the memory rehearsal had a significant indirect effect on Revisit intention through place attachment (*B* = 0.1130, Boot CI: 0.0582–0.1757). Memory rehearsal had a significant indirect effect on recommendation intention through place identity (*B* = 0.0793, Boot CI: 0.0402–0.1258). Memory impact had a significant indirect effect on the Revisit intention through place identity (*B* = 0.1503, Boot CI: 0.0865–0.2277). Memory impact has a significant indirect effect on recommendation intention through place identity (*B* = 0.1002, Boot CI: 0.0472–0.1569). Therefore, H3a–H3d is supported.

## Conclusion and Discussion

### Conclusions of the Study

Based on the theory of autobiographical memory in cognitive psychology, this paper attempts to propose a conceptual model to explain the effect of tourist autobiographical memory on their post-travel behavioral intentions in the context of rural tourism. These paths indicated that the memory of the tourism experience is the antecedent of tourist post-travel behavioral intention, and the two dimensions of autobiographical memory structure (memory rehearsal and memory impact) are important antecedents of revisit intention and word-of-mouth recommendation intention. In particular, place attachment mediates the relationship between autobiographical memory and post-travel behavioral intentions.

### Theoretical Contribution

The first theoretical contribution of this paper is to confirm the Tourism Autobiographical Memory Scale (TAMs) developed by Jorgenson et al. (2019) is supported in the context of rural tourism in China. Based on the perspective of cognitive psychology, according to the structural dimension of autobiographical memory, the scale is divided into two dimensions, namely, memory rehearsal and memory impact. This study confirmed the validity of the structure of the scale, and the scale has a good universality.

Second, previous studies have applied autobiographical memory to memorable tourism experiences (Kim and Jang, 2014; Kim and Chen, 2019). Based on cognitive psychology, this study attempts to propose a causality model between tourist autobiographical memory, place attachment, and post-travel behavior intention. Through the response path of “memory-emotion-intention,” it is clear to explain how the autobiographical memory affects their place attachment and behavioral intention. As far as we know, this is the first empirical study involving the structure of tourist autobiographical memory, which helps to expand and conceptually link cognitive psychology with tourism, and thus innovate through interdisciplinary views (Shen et al., 2019). Furthermore, this study not only expands the applicability of the concept of autobiographical memory in tourism destination research and fills in the literature gap, but also provides a theoretical basis for studying how autobiographical memory affects decision-making. In addition, the existing literature on memory rehearsal is very inadequate. The frequency of memory rehearsal also had a positive effect on behavioral intention, which was another important finding.

Third, previous studies have confirmed that personal past experiences may contribute to a person’s decision on future behavior (Lam and Hsu, 2006), and autobiographical memory has a significant influence on his revisit intentions (Zhang et al., 2020; Zhou et al., 2020). The conclusion of this study supports this argument. In addition, this study also confirms that autobiographical memory significantly affects recommendation intention. It is further suggested that autobiographical memory can greatly affect tourist stickiness, and is an important predictor of the recommendation intention. Therefore, this study not only provides evidence of the importance of autobiographical memory as the antecedent variable of post-travel behavioral intention, but also advances the research on tourist future behavior. These results contribute to the understanding of the antecedents of post-travel behavioral intention.

Finally, previous studies have found that place attachment plays an intermediary (a mediating) role between tourism attitude (Prayag and Ryan, 2012), tourism motivation (van Riper et al., 2019), tourism image (Song et al., 2017), and behavior intention. However, autobiographical memory is rarely regarded as the influencing factor of place attachment. In this study, we found that autobiographical memory can also be used as a prevariable of place attachment, and place attachment plays a mediating role in the relationship between memory rehearsal, memory impact, and post-travel behavioral intention. It also elaborates the relationship between autobiographical memory and place attachment, recommendation intention, and revisit intention. This finding complements existing research on place attachment.

### Practical Implications

First of all, results indicate that autobiographical memory of travel experience is the antecedent of post-travel behavioral intention (including revisit intention and recommendation intention). Therefore, rural tourism destinations managers should attach importance to the tourist’s autobiographical memory and tourist experience (Zhang et al., 2018). The practices of the marketing and management need to shift from product oriented to experience oriented. Kim and Ritchie (2014) point out that people remember an experience that is personal and closely related to their interests, rather than an irrelevant experience. Other studies have shown that only memorable experiences can influence the decision-making process of tourists in the future (Kim et al., 2010b). Therefore, destination managers should attach importance to developing differentiated products and designing tourism projects that tourists actively participate in Kim and Ritchie (2014). At the same time, it provides tourists with unique and personalized tourism products to create travel experiences that more memorable and easier to recall for them (Kim et al., 2010a; Neuhofer et al., 2012, 2015). According to our research, the higher the frequency of memory rehearsal, the stronger revisit intention and recommendation intention are, indicating that the autobiographical memory of the relevant place needs to be repeated from time to time, and the higher the frequency of rehearsal, the stronger the place attachment, revisit intention and recommendation intention are. Some studies have shown that posting personal events online provide opportunities for rehearsals and significance to construct (Wang, 2013), as a result, in addition to provide souvenirs, photos, and other tangible items to arouse tourist’s autobiographical memory, rural tourism destinations can also encourage tourists to actively write travel notes and share their travel experiences with others to improve the frequency of memory rehearsal. Our research also shows that the greater an individual’s memory impact is, the stronger the revisit intention and recommendation intention are. Therefore, rural tourism destinations should identify which types of experiences have the greatest impact on tourists, and constantly optimize and enhance these experiences.

Second, our results suggest that place attachment plays a mediating role between autobiographical memory and tourist post-travel behavioral intentions. Specifically, the stronger the autobiographical memory formed by tourists in the process of tourism, the higher the degree of attachment to the tourism destination, the greater the probability of revisiting, and willing to recommend to their relatives and friends, forming a “word-of-mouth effect.” Therefore, rural tourism destinations managers and marketers should rely on rural localities to develop unique tourism products, establish and promote “memory points” in different locations of the destination (Tung and Ritchie, 2011), and enhance the frequency of memory rehearsal. In addition, storytelling shapes people’s memories and impressions of events (McGregor and Holmes, 1999), and a good performance experience can enhance tourist memories of the destination (Oh et al., 2007). Thus, it is possible to design appropriate story content and themes (Pine and Gilmore, 1998; Tung and Ritchie, 2011) and stage performances for rural tourism destinations enable people to constantly rehearse their memory in the process of listening to and sharing stories, so as to enhance their memory and make it easier to recall. In addition, our results further suggest that memory rehearsal and memory impact have a significant effects on place attachment, with memory impact having a stronger effect on place attachment than memory rehearsal. Therefore, rural tourism destinations managers should constantly excavate local characteristics, deepen tourist understanding of rural tourism destinations, strengthen, and enhance tourist destination attachment level (Wang et al., 2020), and finally establish a sense of place identity. More importantly, since the theory of place attachment is based on people’s feelings, tourism destinations should provide tourists with tourism projects to experience local culture and lifestyle, so that they can have a deeper understanding of local history and culture, so as to further increase their connection with their destination and the significance of their destination to them.

Finally, in the tourism-as-experience paradigm, tourists not only need to gaze at the distant places as “others” to gain visual experience, but also need “dialogue” to understand the meaning and values behind rural localities. Intercultural communication requires not only gazing, but also the dialogue between tourists and hosts. By increasing and improving the interaction between tourists and hosts, we can better understand the local culture and exert and strengthen the memory impact of a place on tourists.

### Research Limitations and Prospects

There are still some limitations in this paper, which need to be further explored in the following research. First of all, while this study develops the theoretical model to investigate the relationship among autobiographical memory, place attachment, and post-travel behavioral intention, which is helpful to understand the causal relationship among these constructs, more research is needed to further validate and extend these concepts. Tourist memory of destination includes not only autobiographical memory, but also semantic memory. Whether semantic memory can also influence tourist behavior needs further examination. Moreover, this study investigates tourist post-travel behavior intentions rather than behaviors, so the actual behaviors should be further measured in future studies. Second, this paper only focuses on two dimensions of autobiographical memory, which can further expand the application of other dimensions of autobiographical memory in tourism research. At the same time, other factors (such as the frequency of visits in the past, the type of tourists, etc.) should be considered to further determine the boundary conditions of the model. Finally, this study only investigates Chinese tourists in rural tourism destinations in China. In order to make the results universal, the model should be tested in other tourism contexts in the future.

## Data Availability Statement

The original contributions presented in the study are included in the article/supplementary material, further inquiries can be directed to the corresponding author/s.

## Ethics Statement

The studies involving human participants were reviewed and approved by School of Tourism, Hainan Normal University. Written informed consent to participate in this study was provided by the participants’ legal guardian/next of kin.

## Author Contributions

ZZ: formulation of research questions, conceptualization, and data collection and analysis (contributed 40%). ZL: literature review, discussions and implications, and manuscript proofreading (contributed 40%). CC: conceptualization and interpretation of results (contributed 20%). All authors contributed to the article and approved the submitted version.

## Conflict of Interest

The authors declare that the research was conducted in the absence of any commercial or financial relationships that could be construed as a potential conflict of interest.

## Publisher’s Note

All claims expressed in this article are solely those of the authors and do not necessarily represent those of their affiliated organizations, or those of the publisher, the editors and the reviewers. Any product that may be evaluated in this article, or claim that may be made by its manufacturer, is not guaranteed or endorsed by the publisher.
